# Effects of 1,25(OH)_2_D_3_ on Cancer Cells and Potential Applications in Combination with Established and Putative Anti-Cancer Agents

**DOI:** 10.3390/nu9010087

**Published:** 2017-01-23

**Authors:** Mohamed A. Abu el Maaty, Stefan Wölfl

**Affiliations:** Institut für Pharmazie und Molekulare Biotechnologie (IPMB), Universität Heidelberg, Im Neuenheimer Feld 364, 69120 Heidelberg, Germany; abu.el.maaty@gmail.com

**Keywords:** 1,25-dihydroxyvitamin D_3_, combination chemotherapy, anti-cancer effects

## Abstract

The diverse effects of 1,25-dihydroxyvitamin D_3_ (1,25(OH)_2_D_3_), the bio-active form of vitamin D, on cancer cell metabolism and proliferation has made it an interesting candidate as a supporting therapeutic option in cancer treatment. An important strategy in cancer therapy is the use of combination chemotherapy to overcome drug resistance associated with numerous anti-cancer agents and to provide better means of avoiding undesirable side effects. This complex strategy is widely adopted by oncologists and several established “cocktails” of chemotherapeutics are routinely administered to cancer patients. Among the principles followed in designing such treatment regimens is the use of drugs with different mechanisms of action to overcome the issue of tumor heterogeneity and to evade resistance. In light of the profound and diverse effects of 1,25(OH)_2_D_3_ reported by in vitro and in vivo studies, we discuss how these effects could support the use of this molecule in combination with “classical” cytotoxic drugs, such as platins and anti-metabolites, for the treatment of solid and hematological tumors. We also examine recent evidence supporting synergistic activities with other promising anti-cancer drug candidates, and postulate mechanisms through which 1,25(OH)_2_D_3_ may help evade chemoresistance.

## 1. Introduction

Several combinations of chemotherapeutics have demonstrated tremendous therapeutic efficacy compared to single-drug treatments, such as the combination of cyclophosphamide, methotrexate, and 5-flurouracil, abbreviated “CMF” for advanced breast cancer, and the “FOLFOX” regimen, comprising 5-flurouracil, folinic acid and oxaliplatin for combating colorectal cancer [1]. This approach, first introduced through the pioneering work of Emil Frei and others in the 1960s [2], remains the backbone for current treatment regimens. Tumor heterogeneity, side effects, and drug resistance that arise from single-drug treatments provide the basis for oncologists to pursue combination therapies [1]. Using drugs of differing mechanisms of action addresses the tumor heterogeneity issue, and augments the efficacy and minimizes the side effects of single treatment modalities, making the further establishment of more combinations clinically attractive. 

Tumor suppression and regulation of cellular proliferation are among a growing list of extra-skeletal effects attributed to vitamin D [3]. Clinical trials designed to evaluate this promising potential are currently underway, whereas observational and experimental data have already provided compelling evidence supporting the association [3,4]. It is plausible that the international guidelines of vitamin D supplementation might be reevaluated upon completion of current trials and optimized for the prevention and treatment of chronic diseases including cancer, given the broad safety margin, price, and availability of the molecule to consumers.

Vitamin D undergoes a two-step metabolic activation leading to the generation of 1,25-dihydroxyvitamin D_3_ (1,25(OH)_2_D_3_), the hormonally active form [4]. 1,25(OH)_2_D_3_ appears to possess a broad-spectrum of anti-tumor effects, albeit in supra-physiological concentrations, which possibly lead to hypercalcemia. To avoid the potential setback associated with such doses, different analogs of the molecule shown to be less calcemic have been developed, such as EB 1089 (seocalcitol) and 22-oxacalcitriol (maxacalcitol) [5,6]. The former, despite profound anti-neoplastic effects on different in vitro and in vivo models, is not clinically approved, whereas the latter is clinically approved in Japan for the treatment of secondary hyperparathyroidism and psoriasis (for a comprehensive overview of clinically approved and experimental vitamin D analogs, the readers are referred to review [7]). As a seco-steoid, 1,25(OH)_2_D_3_ binds to the nuclear vitamin D receptor (VDR), inducing its hetero-dimerization with retinoid X receptor (RXR), and subsequent binding to target genes harboring vitamin D response elements [3,4], ultimately leading to the regulation of hundreds of genes [8]. In respect to cancer, studies have shown that 1,25(OH)_2_D_3_ induces the expression of anti-proliferative and pro-apoptotic genes such as p21, p27, and BAX, and decreases the expression of oncogenic transcription factors like MYC and Hypoxia-Inducible factor 1-α (HIF1α) [3].

In light of our current understanding of the multi-faceted, unconventional roles vitamin D is now known to play, we examined the available evidence indicating additive or synergistic effects of the molecule with classical cytotoxic agents, thereby supporting its use in cancer combination therapy. Due to the high abundance of published data on this theme, we drew special attention to combinations that have been tested on different models and hence reported in a number of publications. Our initial literature search narrowed the focus of this review to combinations of 1,25(OH)_2_D_3_, and its analogs, with: (1) *anti-metabolites* (e.g., 5-fluorouracil and gemcitabine); (2) *platinum compounds* (e.g., cisplatin, oxaliplatin, and carboplatin); (3) *taxanes* (e.g., paclitaxel and docetaxel); and finally (4) *tyrosine kinase inhibitors* (e.g., gefitinib and erlotinib). We therefore provide an overview of the known mechanisms of action of the aforementioned classes of drugs, highlight the additive/synergistic roles 1,25(OH)_2_D_3_ could play in the reported settings, and hypothesize on additional mechanisms through which this molecule could potentiate the effects of conventional cancer chemotherapy. In the end, we shed light on the potentially advantageous combination of 1,25(OH)_2_D_3_ with non-classical anti-cancer agents, such as metformin and auranofin. Promising combinations of vitamin D with non-classical anti-cancer agents also include different phytochemicals such as carnosic acid [9], curcumin [10], and genistein [11], however, these reports are not discussed in length in this review.

### 1.1. 1,25(OH)_2_D_3_ in Combination with Anti-Metabolites

In 1948, Sidney Farber and colleagues reported that “poisoning” cancer cells with aminopterin, an anti-metabolite, induced temporary remission in children with acute leukemia [2]. This finding gave rise to this important class of anti-cancer agents specifically, and cancer chemotherapeutics generally. Since then, numerous members of this class of drugs have been developed and many are considered vital to cancer chemotherapy. Generally, this class acts by either inhibiting the synthesis of DNA or RNA (such as methotrexate), or by being incorporated into these macromolecules and thereby inhibiting their function (such as 6-mercaptopurine), or by both mechanisms (such as 5-Fluorouracil (5-FU) or gemcitabine) [12,13,14].

Due to limitations in the currently available treatment modalities and the complexity of the disease, pancreatic cancer (PCa) is usually associated with poor prognosis. Gemcitabine is the oncologist’s first choice for the management of the disease despite proving only mildly beneficial in terms of life-extending capabilities [15]. Additionally, resistance to this drug is common in such tumors because of the intricate tumor microenvironment associated, referred to as desmoplastic stroma, which acts as a protective “armor”, shielding the tumor from drugs [16]. In light of this, it is not surprising that different groups have investigated the potential use of VDR analogs in combination with gemcitabine in this setting.

A recent high-profile publication by Sherman et al. [17] utilized different mouse models, including the KPC (K-ras^LSL.G12D/+^; p53^R172H/+^; Pdx-1-Cre) model, which resembles human pancreatic tumors in terms of responsiveness to gemcitabine, and showed that the VDR activator—calcipotriol—reverses gene signatures associated with the cancer-promoting, activated pancreatic stellate cells, as well as enhances tumor vascularization and gemcitabine intra-tumoral delivery, leading to an overall reduction in tumor volume. Similarly, Yu et al. [18] illustrated that calcitriol enhances gemcitabine’s anti-tumor effects in both the PCa cell line Capan-1 and in mice bearing Capan-1 tumors, through the activation of caspases-8, 9, 6, and 3, and the inhibition of the pro-survival signaling molecule Akt.

Conversely, Bhattacharjee et al. [19] performed a genome-wide siRNA screen on the PCa cell line Panc1 and demonstrated that loss of the VDR gene sensitizes cells to gemcitabine treatment, and that knocking down of the gene led to profound reductions in the drug’s IC_50_ values in two other PCa cell lines. Furthermore, the authors showed that cell line VDR expression correlated with gemcitabine resistance, and that survival of cells after stable knock down of the VDR gene was only observed in one cell line, illustrating the gene’s importance for PCa cell survival.

5-FU, as previously mentioned, inhibits both the synthesis and function of nucleic acids. Intracellularly, 5-FU undergoes different metabolic steps that ultimately determine its anti-tumor mode of action. On one hand, it is metabolized to fluorouridine triphosphate, which gets incorporated into RNA molecules, thereby inhibiting their function [13]. On the other hand, 5-FU is metabolized to fluorodeoxyuridine diphosphate, which is further metabolized to either fluorodeoxyuridine monophosphate or fluorodeoxyuridine triphosphate. The former inhibits thymidylate, and subsequently DNA synthesis, by binding to and inhibiting thymidylate synthase [13]. The latter, however, gets incorporated into DNA molecules and inhibits their functions [13].

In spite of its effectiveness against different tumors, it is believed that 5-FU’s success is most profound in colorectal cancer [13]. It is therefore not surprising that several groups have investigated the combination of 1,25(OH)_2_D_3_ with 5-FU in different colorectal cancer models. Using an azoxymethane-induced colorectal cancer rat model, El-Shemi et al. [20] and Refaat et al. [21] demonstrated that the combination of paricalcitol (a synthetic VDR analog), in case of the former, and vitamin D_3_, in case of the latter, together with 5-FU, led to significant reductions in the number of tumors grown. Additionally, both studies illustrated enhanced interference of this combination with the Wnt signaling pathway, through decreasing the expression of Wnt and β-catenin, and the induction of the pathway’s inhibitor, Dkk1. Similarly, Milczarek et al. [22] observed that the combination of 5-FU with either vitamin D analog, PRI-2191 or PRI-2205, inhibits tumor growth and metastasis as well as prolongs survival in MC38 colon cancer cell-bearing mice.

### 1.2. 1,25(OH)_2_D_3_ in Combination with Platinum Compounds

Another landmark in cancer chemotherapeutics is the serendipitous discovery of the cytotoxic effects of *cis*-diamminedichloridoplatinum(II) (cisplatin or CDDP) in Barnett Rosenberg’s lab in the 1960s [23]. Subsequent investigations utilizing a series of platinum compounds, including cisplatin, demonstrated their potent effects on sarcoma and leukemia mouse models [24]. Cisplatin covalently binds to DNA and leads to the inhibition of proliferation and DNA damage mediated cell death [23], which is particularly effective in rapidly dividing tumor cells. Less than a decade later, cisplatin received Food and Drug Administration (FDA) approval and made its way to the clinic [23]. Despite this being a major breakthrough in the field, patients treated with cisplatin suffered its infamous side effects, mainly nephro- and neuro-toxicity, which encouraged the pursuit of new and improved platins, namely carboplatin [23]. Besides side effects, another substantial obstacle was cisplatin-resistance exhibited by certain tumors, thought to be mediated by either reduced drug uptake, detoxification in the cytoplasm, or increased cellular export [23]. This led to the development of oxaliplatin, a component of the aforementioned FOLFOX regimen, as well as other new generation platins [23].

Investigations of VDR activator-platin combinations date back to the early 1990s, and are still a recurring theme in the literature. 1,25(OH)_2_D_3_ and its analogs have been combined with different platins and tested on different in vitro and animal models with mostly positive results. Cisplatin binds covalently to DNA forming adducts, which induces cell cycle arrest and apoptosis [23], a mechanism that has been shown to be potentiated by 1,25(OH)_2_D_3_. Using in vivo and in vitro models of squamous cell carcinoma, Ma et al. [25] showed that 1,25(OH)_2_D_3_ sensitizes cells to cisplatin-induced cytotoxicity and apoptosis. The authors went on to demonstrate that the induction of p73 by 1,25(OH)_2_D_3_ is crucial for the observed effects, since knocking down of the p73 gene blunted potentiation. Prior to this publication, the same group showed that in the same in vitro model, 1,25(OH)_2_D_3_ and cisplatin exhibit distinct molecular effects, such as opposing regulation of p53 and its target genes [26]. Based on their investigations, they proposed that the enhanced apoptotic effect observed in combination treatment could be due to a synergistic upregulation of mitogen activated protein kinase kinase kinase 1 [26].

Similarly, Jorgensen et al. [27] showed that 1,25(OH)_2_D_3_ enhanced cisplatin’s cytotoxic effects in NTera2, an embryonal carcinoma-derived cell line. However, these additive effects were not observed in an NTera2 xenograft model. Underlying mechanisms of this combination in both systems were shown to involve an upregulation of p21, p27, p53, p73, and FOXO1, and a decrease in the OCT4 pluripotency gene [27]. Also, Kulkarni et al. [28] demonstrated beneficial effects of combining 1,25(OH)_2_D_3_ with cisplatin in a human retinoblastoma xenograft model.

Furthermore, combinations of 1,25(OH)_2_D_3_ analogs with platins have also spawned positive results. Pelczynska et al. [29] tested the combination of two 1,25(OH)_2_D_3_ analogs, PRI-2191 and PRI-1906, with a number of cytostatic drugs, and observed a significant decrease in IC_50_ values of cisplatin when combined with the analogs in a number of cell lines. Additionally, Milczarek et al. [30] showed that the combination of oxaliplatin with 1,25(OH)_2_D_3_ analogs enhanced the therapeutic potential in a mouse model bearing the human colorectal cancer cells HT-29, in terms of tumor volume reduction, however, antagonism was observed in combination therapy, compared to mono-treatment with oxaliplatin, with regards to mean survival time.

In SKOV3 ovarian cancer cells, Zhang et al. [31] demonstrated an induction of reactive oxygen species and apoptosis, as well as cell cycle arrest upon treatment with a combination of carboplatin and 1,25(OH)_2_D_3_.

### 1.3. 1,25(OH)_2_D_3_ in Combination with Taxanes

Paclitaxel (Taxol) was originally discovered in the 1960s as part of a National Cancer Institute screening program of plant extracts for anti-tumor effects [32]. Taxanes (name derived from the plant genus *Taxus*) have been shown to be effective against early and late-stage cancers, and work by binding to microtubules, preventing their normal functions [33].

Several studies have reported enhanced cytotoxic effects upon combing 1,25(OH)_2_D_3_ with different taxanes compared to single drug treatments. For example, Wang et al. [34] reported that pretreatment of breast cancer cells with 1,25(OH)_2_D_3_ followed by paclitaxel significantly reduced the EC_50_ of the latter. Similarly, in both in vitro and in mice bearing either human prostate or murine squamous cell carcinomas, Hershberger et al. [35] demonstrated that pretreatment with 1,25(OH)_2_D_3_ enhanced the growth inhibiting effect of paclitaxel. Moreover, Ting et al. [36] illustrated that pretreatment of prostate cancer PC3 cells with 1,25(OH)_2_D_3_ enhanced docetaxel’s anti-cancer effects and increased the percentage of apoptotic cells. The authors also demonstrated that 1,25(OH)_2_D_3_ decreased mRNA and protein expression of multidrug resistance-associated protein-1, and subsequently concluded that 1,25(OH)_2_D_3_ may enhance docetaxel’s effects through decreasing the level of this protein.

### 1.4. 1,25(OH)_2_D_3_ in Combination with Tyrosine Kinase Inhibitors

Tyrosine kinases represent a newer class of druggable targets compared to the more established targets of cytotoxic chemotherapy. Enhanced activation of these enzymes, whether due to somatic mutations or growth factor signaling, leads to increased growth, proliferation, and angiogenesis, as well as evasion of apoptosis [37]. A handful of drugs shown to target both receptor- and non-receptor tyrosine kinases have demonstrated great potential in pre-clinical and clinical trials, with the lead drug imatinib (STI571; Gleevec) already being administered to patients with chronic myelogenous leukemia harboring the Philadelphia chromosome, as well as c-KIT positive, gastrointestinal stromal tumor patients [37].

Two independent studies have reported that the combined use of 1,25(OH)_2_D_3_ with different tyrosine kinase inhibitors (TKIs)—gefitinib/erlotinib (epidermal growth factor receptor (EGFR) inhibitors) and sunitinib (multi-target TKI)—induces differentiation of acute myeloid leukemia cells. Nishioka et al. [38] showed that the combination of sunitinib and 1,25(OH)_2_D_3_ enhances the expression of CD11b and the production of both interferon γ and tumor necrosis factor α. Similarly, Lainey et al. [39] showed that either of the aforementioned EGFR inhibitors, together with 1,25(OH)_2_D_3_, induces markers and processes associated with differentiation, such as CD11b and CD14 expression, cell cycle arrest, apoptosis, and NADPH oxidase activity. Moreover, a recent study investigating the potential use of erlotinib together with 1,25(OH)_2_D_3_ in head and neck squamous cell carcinoma utilized in vivo models to demonstrate augmented reduction of tumor growth in the combination regimen compared to single drug treatments [40]. Further analyses illustrated that this combination reduces phosphorylation of Akt (S473) and EGFR (Y1092) [40].

Despite profound potency and lack of major side effects, initial or acquired resistance to TKIs remains an issue. Resistance to imatinib, for example, has been shown to be mediated through various mechanisms, such as mutations in the kinase domain of BCR-ABL, as well as re-activation and amplification of the oncogene [37]. It remains unclear if and how 1,25(OH)_2_D_3_ may aid in evading this resistance, however, results of previous studies on different combinations warrant further investigations into this theme.

### 1.5. 1,25(OH)_2_D_3_ in Combination with Non-Classical Anti-Cancer Drugs

The long and costly path of drug discovery and development has encouraged researchers to seek additional roles for drugs that have already been approved, a process known as drug repurposing. Metformin and auranofin are two promising examples of such drugs, and are currently being evaluated for use in cancer chemotherapy.

**Figure 1:**
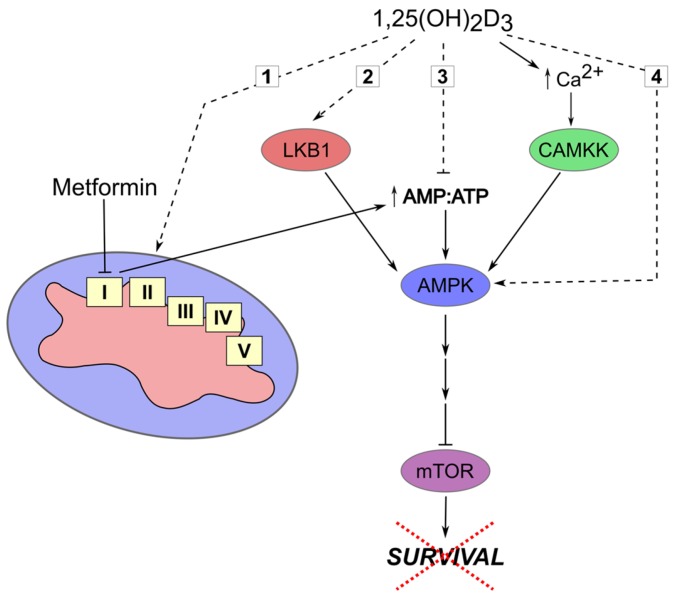
Dual targeting of AMPK signaling by 1,25(OH)_2_D_3_ and metformin. Hypothesized mechanisms through which 1,25-dihydroxyvitamin D_3_ (1,25(OH)_2_D_3_) influences the activity of AMP-activated protein kinase (AMPK) and hence cell survival are depicted by dashed lines and include: (1) Modulation of mitochondrial biogenesis/activity with the subsequent deregulation of energy status; (2) Induction of LKB1 expression/activity; (3) Disruption of non-mitochondrial energy-producing processes (e.g., cellular glucose uptake and glycolysis); (4) Direct AMPK activation via induction of AMPK expression or inhibition of inactivating phosphatase expression.

In 2005, Evans et al. [41] observed that among type 2 diabetics, subjects treated with metformin had a lower risk of developing cancer. Since then, several reports have used in vitro and animal models to dissect metformin’s anti-cancer mechanism of action, and different clinical trials are underway [42]. It is now understood that metformin is an AMP-activated protein kinase (AMPK) indirect activator that works by inhibiting complex 1 of the electron transport chain, leading to an increase in AMP:ATP ratio, which in turn activates AMPK signaling [42]. Activation of this pathway elicits varied biological responses, including inhibition of the pro-survival signaling pathway, mammalian target of rapamycin (mTOR) [42]. Furthermore, AMPK’s tumor suppressor role is largely strengthened by its activation by another well characterized tumor suppressor, Liver-kinase B1 (LKB1) [43]. Besides this kinase, an additional upstream kinase regulates the activity of AMPK, namely calcium/calmodulin-dependent protein kinase kinase (CAMKK) [43]. This molecule is activated by an increase of intracellular calcium levels, and subsequently activates AMPK [43]. It is therefore plausible that 1,25(OH)_2_D_3_, given its established calcium homeostasis regulating role, activates AMPK via inducing CAMKK activity. Alternatively, 1,25(OH)_2_D_3_ may increase the AMP:ATP ratio via influencing different energy producing pathways, for example, through modulation of mitochondrial biogenesis/activity, or inhibition of glycolysis or beta-oxidation of fatty acids, which would in turn activate AMPK (Figure 1).

Several studies have investigated the potential of the vitamin D-metformin combination using in vitro models of breast [44], prostate [45], and bladder cancer [46], demonstrating enhanced anti-cancer effects in all reports. Furthermore, Li et al. [47] utilized two different in vivo models of colorectal cancer to investigate the efficacy of this combination and illustrated a significant decrease in the number of tumors and aberrant crypt foci in colons of animals treated with the combination compared to either drug alone. The proposed mechanism of action involved influencing the AMPK-mTOR axis.

Auranofin, on the other hand, is a disease-modifying anti-rheumatic drug (DMARD) initially used to treat rheumatoid arthritis, however now known to possess growth-inhibiting properties in a number of experimental models [48,49]. It is the lead structure for gold-complexes, which exert anti-tumor effects by inhibiting their intracellular target, thioredoxin reductase (TrxR), thereby inducing oxidative stress and apoptosis [48,49].

**Figure 2:**
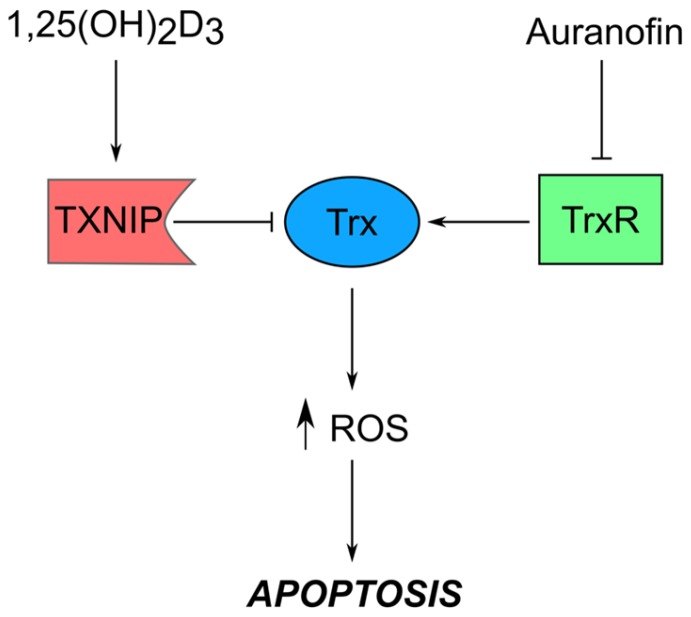
Potential cooperative targeting of the thioredoxin system by 1,25(OH)_2_D_3_ and auranofin. Induction of thioredoxin-interacting protein (TXNIP)/ vitamin D3-upregulated protein 1 (VDUP1) expression with 1,25(OH)_2_D_3_ and inhibition of thioredoxin reductase (TrxR) by auranofin and other gold complexes could synergistically inhibit the reductive capacity of the thioredoxin (Trx) system, subsequently increasing intracellular reactive oxygen species (ROS) levels, which activate apoptotic signaling. Besides influencing redox balance, 1,25(OH)_2_D_3_-mediated TXNIP induction may alter glucose homeostasis given the latter’s role in regulating intracellular glucose levels. Although this effect is independent of other players of the Trx system (hence not presented in figure), it could define novel anti-tumor roles of 1,25(OH)_2_D_3_.

TrxR is a member of the thioredoxin (Trx) system which plays an important role in maintaining intracellular redox balance. TrxR reduces Trx, which in turn acts as a protein disulfide reductase [50]. Thioredoxin-interacting protein (TXNIP) is another member of the Trx system, and works by binding to, and subsequently inhibiting the activity of, reduced Trx [51]. Noteworthy is that TXNIP was originally identified by Chen and Deluca in 1994 as the vitamin D3-upregulated protein 1 (VDUP1) through screening of regulated cDNAs in response to 1,25(OH)_2_D_3_ treatment of HL-60 leukemia cells [52]. Since then, besides being characterized as Trx’s binding partner, studies have shown that TXNIP is a major regulator of glucose homeostasis that works by sensing the levels of glucose and glycolytic intermediates, and reduces glucose influx in response to increased intracellular levels [53]. Furthermore, TXNIP expression is controlled by various transcriptional regulators like MondoA/MLX [53], MYC [54], and HIF1α [55], and is shown to be silenced in many cancers [51]. We therefore hypothesize that dual targeting of the Trx system using a TrxR inhibiting gold complex, as well as 1,25(OH)_2_D_3_, a potential TXNIP inducer, may synergistically induce oxidative stress in cancerous cells, which would essentially trigger pro-apoptotic signaling (Figure 2). To our knowledge, this combination has only been reported once, not from a Trx system perspective, but rather a differentiation one, where the authors of the study reported that the 1,25(OH)_2_D_3_-auranofin combination synergistically induced differentiation of HL-60 cells [56].

### 1.6. Overcoming Chemoresistance with 1,25(OH)_2_D_3_

The major setback hampering initial success of many chemotherapeutics is resistance acquired by tumors during treatment. Imposed therapeutic pressure induces cancerous cells in many cases to adopt one or more well-known mechanisms of resistance (Table 1), such as reduced drug uptake due to altered expression of transporters, reduced activation/enhanced inactivation of drugs, and increased drug efflux, through up-regulation of, most commonly, ATP binding cassette (ABC) transporters [57]. It is believed that selected cells originate from either cancer stem cells (CSCs), which have profound tumor-initiating properties, or through support of the tumor microenvironment, via providing growth factors and cytokines capable of inducing a resistance-promoting transcriptional program [57].

The ability of 1,25(OH)_2_D_3_ to overcome tumor stroma-mediated chemoresistance has been elaborately shown, as previously described, in the case of PCa [17]. Similarly, the ability of 1,25(OH)_2_D_3_ to influence major signaling pathways associated with CSCs, namely Transforming growth factor-β (TGF-β), Wnt, Notch, and Hedgehog signaling, has been previously demonstrated and comprehensively reviewed in [62].

**Figure 3:**
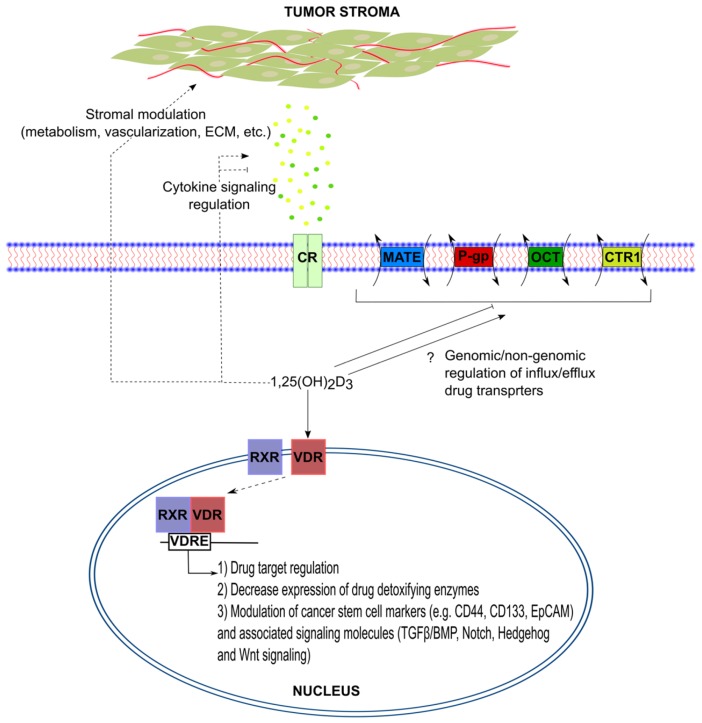
Overcoming chemoresistance with 1,25(OH)_2_D_3_. Through genomic or non-genomic mechanisms, 1,25(OH)_2_D_3_ may affect the various players involved in mediating resistance to chemotherapeutics, such as modulating tumor microenvironment, cancer stem cell signaling, as well as drug influx, efflux, and metabolism.

The possibility however, that 1,25(OH)_2_D_3_ influences drug influx/efflux has not been thoroughly investigated. Given the wealth of literature on the synergism between 1,25(OH)_2_D_3_ and cytotoxic drugs, it is conceivable that these effects could be at least partly explained by 1,25(OH)_2_D_3_ setting the “transcriptomic scene” for the drugs to operate more effectively (Figure 3). In other words, 1,25(OH)_2_D_3_ may normalize the expression of chemoresistance mediators, such as the drug transporters P-gp, CTR1, and OCT1, thereby enhancing drug influx and reducing efflux. Although 1,25(OH)_2_D_3_ alone possesses anti-cancer effects, we postulate that in settings where tumors show either subtle or no growth inhibition in response to 1,25(OH)_2_D_3_ but are strongly impacted by a combination of 1,25(OH)_2_D_3_ and a cytotoxic drug, the aforementioned hypothesis may come into play.

## 2. Conclusions

The available evidence strongly supports the use of 1,25(OH)_2_D_3_ in cancer chemotherapy and prevention. The advantages of incorporating this molecule into already existing combination regimens include the broad safety profile, low cost, and minimal side effects associated with its use. Furthermore, we postulate that combining 1,25(OH)_2_D_3_ with repurposed drugs (e.g., metformin and auranofin) may significantly enhance their efficacy. We finally put forth the possibility that 1,25(OH)_2_D_3_ may enhance the activity of cytotoxic drugs by increasing their intracellular availability.

## Figures and Tables

**Table 1:** Common resistance mechanisms observed with different chemotherapeutics. Molecular aberrations leading to resistance to the various classes of chemotherapeutics are classified as either influencing drug influx/efflux, metabolism, or mutations of target.

Drug	Resistance Mechanism	References
*Anti-metabolites*		
5-FU	Aberrant expression of:	[13,15]
Gemcitabine	*Thymidylate synthase* *Thymidine phosphorylase* *Dihydropyrimide dehydrogenase* *Human equilibrative nucleoside transporter 1*	
*Platins*		
Cisplatin	Aberrant expression of:	[23]
Carboplatin	*Copper transporter (CTR1)* *ATPase copper transporting alpha (ATP7A)* *ATPase copper transporting beta (ATP7B)* *ATP binding cassette subfamily C member 2 (ABCC2)* *Excision repair cross-complementing-1 (ERCC1)* *mutL homolog 1(MLH1)*	
*Taxanes*		
Paclitaxel	Increased P-glycoprotein (P-gp) expression	[58]
Docetaxel	Altered microtubule dynamics and binding of drug to target	
*TKIs*		
Gefitinib	Mutations in target	[37,59,60,61]
Erlotinib	Induced expression of *MET* and/or *HGF*	
Sunitinib	Aberrant drug influx/efflux (*OCT1* and/or *ABCB1*)	

## Abbreviations

1,25(OH)_2_D_3_	1,25-dihydroxyvitamin D_3_
5-FU	5-Fluorouracil
ABC	ATP binding cassette
AMPK	AMP-activated protein kinase
BMP	Bone morphogenetic protein
CAMKK	Calcium/calmodulin-dependent protein kinase kinase
CMF	Cyclophosphamide—Methotrexate—5-Flurouracil
CR	Cytokine receptor
CSCs	Cancer stem cells
CTR1	Copper transporter 1
FDA	Food and Drug Administration
FOLFOX	5-Flurouracil—Folinic acid—Oxaliplatin
HIF1α	Hypoxia-Inducible factor 1-α
LKB1	Liver-kinase B1
MATE	Multidrug and toxic compound extrusion
mTOR	Mammalian target of rapamycin
OCT	Organic cation transporter
P-gp	P-glycoprotein
PCa	Pancreatic cancer
ROS	Reactive oxygen species
RXR	Retinoid X receptor
TGF-β	Transforming growth factor-β
TKI	Tyrosine kinase inhibitor
Trx	Thioredoxin
TrxR	Thioredoxin reductase
TXNIP/VDUP1	Thioredoxin-interacting protein/vitamin D3-upregulated protein 1
VDR	Vitamin D receptor
VDRE	Vitamin D response elements
